# Long-term observation of glare and dynamic pupil after EVO ICL (implantable collamer lens) for myopia

**DOI:** 10.1186/s12886-025-04377-1

**Published:** 2025-10-09

**Authors:** Xun Chen, Wuxiao Zhao, Jifang Wang, Mingrui Cheng, Xiaoying Wang, Xingtao Zhou

**Affiliations:** 1 Fudan University Eye Ear Nose and Throat Hospital, Shanghai, 200031 China; 2 Key Laboratory of Myopia, Chinese Academy of Medical Sciences, NHC Key Laboratory of Myopia, Fudan University, Shanghai, 200031 China; 3 Shanghai Research Center of Ophthalmology and Optometry, No. 19 BaoQing Road, XuHui District, Shanghai, China; 4 Center for Optometry and Visual Science, Guangxi Academy of Medical Sciences, The People’s hospital of Guangxi Zhuang Autonomous Region, Nanning, 530021 China; 5 Department of Nursing, Eye & ENT Hospital, Fudan University, Shanghai, 200031 China

**Keywords:** Myopia, EVO-ICL, Glare, Pupillometry

## Abstract

**Purpose:**

To investigate the long-term changes in glare and dynamic pupil after EVO ICL implantation in myopic patients and their influencing factors.

**Method:**

In this prospective, consecutive case study, 22 patients (44 eyes, Male/Female = 9/13) underwent EVO-ICL, with a mean age of 25.3 ± 5.4 years and spherical equivalent of -10.52 ± 3.02 D (range: -3.25 D ~ -18.00 D). Comprehensive examinations were performed before and after surgery and all patients were followed up for 5 years.

**Result:**

At the final follow-up, the efficacy and safety indexes were 0.95 ± 0.28 and 1.17 ± 0.19, respectively. The postoperative glare decreased significantly at 1 week and 1 month (*P* < 0.01), then stabilized (*P* > 0.05). 16/22 (72.7%) of the patients reported glare. Of these patients, 7/22 (31.8%) experienced glare bothersomeness, all rating mild. Additionally, 4/22 (18.2%) of the patients had mild problems with night-time driving. In dynamic pupillometry, the amplitude of pupil contraction significantly decreased during early period (*P* = 0.001), and restored after 5 years post-surgery (*P* = 0.081). Moreover, the pupil contraction and dilation velocity reduced significantly in the early-stage (*P* < 0.01), and later recovered after 5 years of surgery (*P* > 0.05). The maximum pupil diameter did not alter significantly in the early-stage, however, it significantly decreased after 5 years post-surgery (*P* = 0.001). GEE showed that pupil dilation velocity, maximum pupil diameter, and ICL size were positively correlated with postoperative glare and were the main influences on the latter (all *P* < 0.01).

**Conclusion:**

EVO ICL is safe and effective in correcting myopia. Post-operative glare is related to the pupil dilation velocity, maximum pupil diameter, and decreases over time. Most patients have no glare bothersomeness in the long term, some patients may even have their glare symptoms disappear. Only a few patients experience slight interference while nighttime driving.

## Introduction

Glare and halos are common nighttime symptoms after implantation of an artificial lens (implantable collamer lens, ICL) in centrally-perforated crystalline lenses, which can affect patient satisfaction and willingness to opt for surgery [1, 2], and have become one of the focuses of attention for both doctors and patients.

Prior single-centre, cross-sectional studies on predictors of glare after ICL indicate that individuals older than 36 years are more prone to experiencing visual symptoms [3]. Additional data indicate that in myopic patients, glare can occur in approximately 15.6–65.2% of cases, and halos can appear in as many as 68.8–93.5% of cases in the early postoperative period (6 months) after ICL [4, 5]. Furthermore, in the long-term postoperative period (19 ± 14 months) after ICL, the prevalence of glare can still be as high as 66.7% and halos 90.1% [3]. The mentioned data, which used the Quality of Vision (QoV) questionnaire, only considered one subjective report of the outcomes of patients, thereby neglecting to reflect the changing glare levels after Implantable Collamer Lens (ICL) surgery in myopic patients.

Previously, our team employed an objective method (Metrovision, France) for continuous monitoring of early post-ICL glare in myopic patients. The results showed that the post-operative glare was relatively stable [5, 6]. However, continuous observation is required for the long-term manifestation of this symptom. To date, only a limited quantity of literature has focused on the analysis of the effects of dynamic pupillometry and ICL parameters on post-ICL glare in myopic patients.

This study aims to measure glare and dynamic pupil objectively and combine subjective symptoms of patients after ICL in myopic patients, in order to examine the long-term characteristics of glare symptoms following ICL implantation and analyze the main factors that influence these symptoms.

## Patients and methods

### Patients

This is a prospective, non-randomized, consecutive case study. The study was conducted in accordance with the Declaration of Helsinki and the Ethics Committee of the Fudan University Eye, Ear, Nose and Throat Hospital, and all patients signed an informed consent form before surgery. Inclusion criteria: ① patients with myopia or myopic astigmatism, corrected visual acuity ≥ 1.0; ② contact lens wearers had stopped wearing soft contact lenses for at least 1 week and hard contact lenses for at least 1 month preoperatively; ③ refractive status was stable (annual change in myopia ≤ 0. 50 D) and preoperative corneal endothelial cell density (ECD) in EVO ICL patients were > 2,000/mm^2^, anterior chamber depth (ACD) ≥ 2.6 mm, white-to-white > 11 mm. Participants with other ocular diseases, history of trauma or surgery, and systemic diseases were excluded.

All participants underwent routine pre-operative examinations including slit lamp biomicroscopy, uncorrected distance visual acuity, intraocular pressure, pupil diameter, axial length, central corneal thickness, Pentacam HR^®^ from Oculus Optikgeräte (Wetzlar, Germany), endothelial cell count, optical coherence tomography (OCT, Optovue, USA), glare test from MonPack One (Metrovision, France), cyclopedic refraction, fundus, and ultrasound biomicroscopy (UBM, Quantel medical, France).

### Dynamic pupillometry

The dynamic pupillometry procedure was similar to that previously described by Zhao et al. [7]. Pupillary Light reflex measurements were started after 15 min of dark adaptation in a dark room. The white Light stimulus delivered during the measurement period was flickering for 200 ms, with a luminance of 100 cd/m^2^, an interstimulus interval of 3300 ms, and a total Light intensity of 20 lx. The average of at least six valid measurements collected within 90 s was used as the dynamic pupil light reflex result for each eye. Dynamic pupil measurement parameters included average response parameters (e.g., initial pupil diameter, amplitude of contraction, latency to contraction, duration of contraction, velocity of contraction, latency to dilation, duration of dilation, velocity of dilation, etc.) and temporal response parameters (maximum, minimum and average pupil diameters). Based on the results of a previous study [7], only the parameters of amplitude of contraction, velocity of contraction and velocity of dilation, and maximum, minimum, and average pupil diameter were extracted for analysis in this study.

### Glare test

The procedure for measuring the halo radius was similar to that previously reported [2]: 200,000 cd/m^2^ white Light Emitting Diodes (LEDs) were built into the left and right sides of the Metrovision display as a glare Light source, the patient was seated at a distance of 2.5 m directly in front of the display, and the monocular test was initiated with the same side of the patient as the Light source was illuminated on one side of the display. The optotype illumination was set to 5 cd/m². The patients were then instructed to read 3 rows of letters - one from each of the top, middle and bottom sections, respectively - which were fan-polygonal to the light source. The patients were asked to read the letters one by one from the side opposite to the light source. For each row of letters, the position of the letters that couldn’t be read because they were covered by the halo radius was recorded, and the mean value of the viewing angle between the unread letters and the light source was calculated as the halo radius and the unit was expressed in minutes of arc. All patients were tested prior to the operation with correction and after the operation without correction.

### Questionnaire

The objective of this questionnaire [8] was to evaluate the frequency, severity, and bothersomeness of glare, and determine their impact on the night driving ability of patients last month. Scores on a 0 to 3 scale, with 0 (none) and 3 (very often, severe, or very bothersome), were used to rate the frequency, severity, and bothersomeness of glare. The level of night-time driving behavior was rated on a 0 to 3 scale: Four driving modes are defined as follows: 0 is normal driving below 120 km/h, 1 is city driving below 80 km/h, 2 is local driving below 40 km/h, and 3 is constrained driving.

### Surgical techniques

The surgical procedure for EVO ICL was similar to that reported by Zhao et al. [2]. The operator inserted the ICL through a 3 mm incision on the temporal side of the cornea and then injected a small amount of viscoelastic into the anterior chamber. The operator adjusted the hook to place the ICL in the posterior chamber and then removed the viscoelastic from the anterior chamber with lactated Ringer’s solution before closing the corneal incision. The ratio of ICL to Toric ICL was 25:19 and all the types of lens were EVO ICLs.

### Postoperative regiment and follow-up

The patient’s intraocular pressure was monitored for 2 to 4 h after surgery, and topical anti-inflammatory and anti-infective treatments were administered: Prednisolone Acetate Ophthalmic Suspension eye drops (Allergan Pharmaceuticals Ireland) 4 times/day for 4 days, moxifloxacin eye drops 4 times/day for 14 days, and pranoprofen eye drops 4 times/day for 14 days; and the use of artificial tears was encouraged 4 times/day until 1 to 3 months after surgery. Follow-up examinations were performed at 1 day, 1 week, 1 month, 3 months and 5 years after surgery.

### Statistical analysis

SPSS 26 software (IBM, USA) was used for statistical processing. Quantitative data were described as “mean ± standard deviation” and qualitative data were described as frequencies and percentages; mixed linear models were used to analyze changes in indicators of postoperative glare, dynamic pupil parameters, ECD, vault, etc. over time after ICL surgery; generalized estimating equations (GEE) were used to investigate the effects of clinical parameters such as residual refraction, uncorrected distance visual acuity (UDVA), dynamic pupil parameters, Ordering parameters for ICL, vault and axial length on postoperative glare. *P* < 0.05 was considered a statistically significant difference.

## Results

### Patients

A total of 22 participants, including 9 male and 13 female, with moderate to high myopia who underwent ICL at the Fudan University Eye, Ear, Nose and Throat Hospital were included. The average age of participants was 25.3 ± 5.4 years (ranging from 18 to 34 years), with a preoperative spherical equivalent of −10.52 ± 3.02 D (ranging from − 18.00 D to −3.25 D). Table 1 presents the preoperative profile of all participants. All patients underwent uneventful surgery and did not experience high intraocular pressure (IOP), ICL adjustment or replacement, or subcapsular opacification of the anterior lens during the follow-up period.

**Table 1:** Preoperative data

Characteristics	mean ± SD/f	Range/%
Age, y	25.3 ± 5.4	18 ~ 34
Sex (M/F)	9/13	40.91/59.09
Sphere, D	−9.73 ± 2.98	−3.00 ~ −17.50
Cylinder, D	−1.59 ± 1.16	0 ~ −4.25
Spherical equivalent, D	−10.52 ± 3.02	−3.25 ~ −18.00
Corrected distance visual acuity, logMAR	−0.073 ± 0.155	−0.1 ~ 0.1
Axial length, mm	27.44 ± 1.18	25.07 ~ 30.35
Central corneal thickness, µm	524.11 ± 35.99	474 ~ 604
Anterior chamber depth, mm	3.22 ± 0.22	2.68 ~ 3.55
K_1_, D	43.61 ± 1.01	39.9 ~ 44.5
K_2_, D	44.28 ± 1.20	41.3 ~ 46.3
White-to-white, mm	11.98 ± 0.31	11.3 ~ 12.6
mesopic pupil size, mm	6.92 ± 0.74	5.1 ~ 8.2
Epithelial cell density, cells/mm^2^	3090.48 ± 251.49	2526 ~ 3653
Intraocular pressure, mmHg	11.98 ± 0.31	11.2 ~ 20.4
Halo radius @ 5 cd/m^2^, arc min	162.73 ± 87.37	60 ~ 330

*logMAR* logarithm of the minimum angle of resolution

### Refractive outcomes

At final follow-up, the efficacy index was 0.95 ± 0.28 and the safety index was 1.17 ± 0.19. Efficacy: 73% with UDVA ≥ 1.0 and 55% with ≥ 1.2. Safety: corrected distance visual acuity (CDVA) improved ≥ 2 Lines from baseline in 18%, improved ≥ 1 Line in 41% and remained unchanged in 39%; only 1 eye (2%) lost 1 line of corrected distance visual acuity and there was no loss of ≥ 2 Lines of corrected distance visual acuity. The mean axial length of the patients was 27.55 ± 1.21 mm, of which 31.8% (14/44) had an increase of > 0.10 mm, and their mean preoperative SE was − 11.26 ± 1.76 D (range: −8.88 D ~ −15.38 D).

**Fig. 1:**
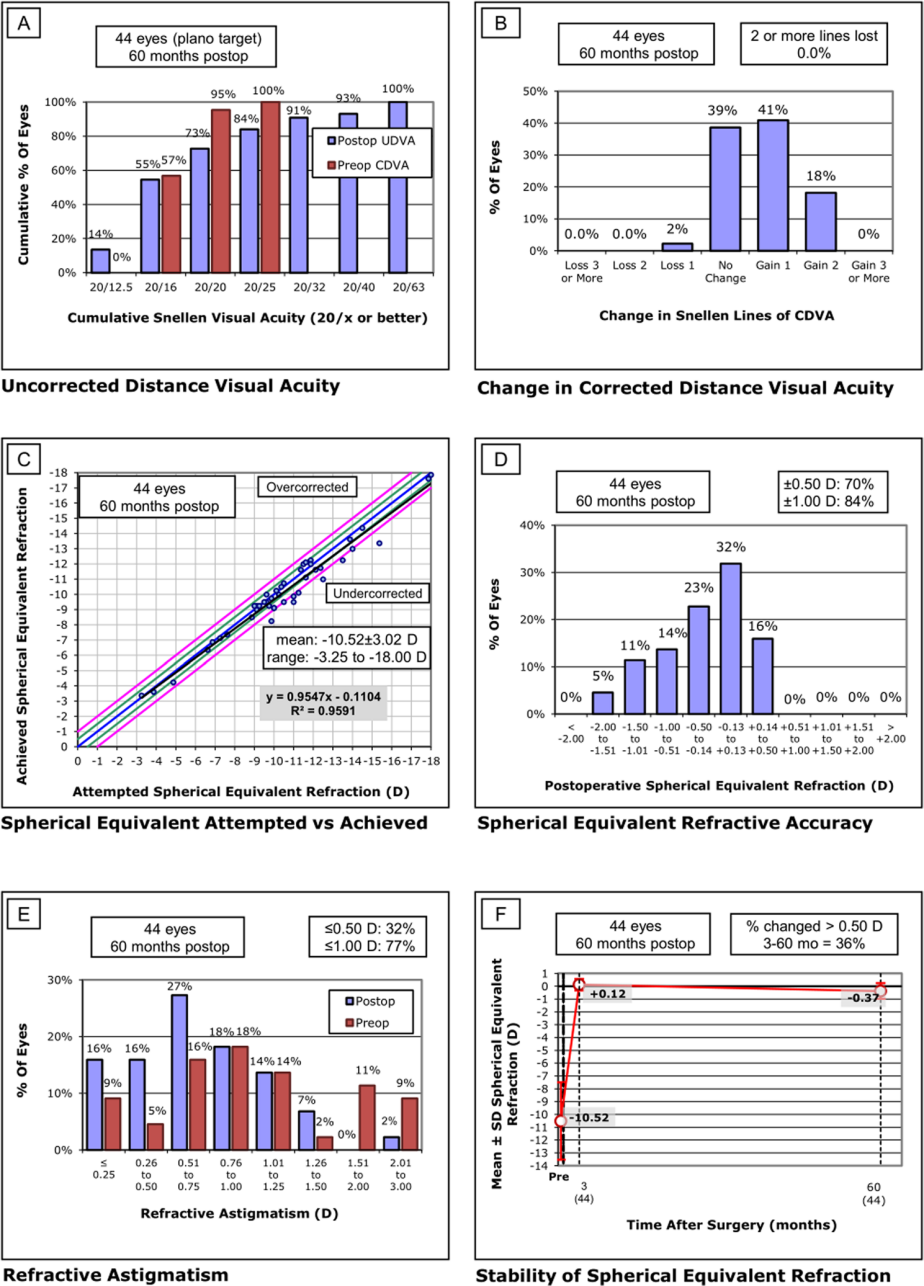
Standard refractive outcomes for patients 5 years after EVO implantable collamer lens (ICL) implantation

The refractive results of all operated eyes are shown in Fig. 1. 100% of eyes with UDVA ≥ 20/63, 93% of eyes with UDVA ≥ 20/40, and 73% of eyes with UDVA ≥ 20/20 (Fig. 1A); 1 eye experienced a loss of 1 Line of corrected visual acuity, but none experienced a loss of 2 or more lines (Fig. 1B). The scatterplot showed good predictability between the proposed and actual corrected refractions (R^2^ = 0.9591, Fig. 1C). The spherical equivalent (SE) of the operated eyes was − 0.37 ± 0.61 D, 71% of the operated eyes were at ± 0.50 D and 84% of the operated eyes were at ± 1.00 D (Fig. 1D). Residual astigmatism was < ± 0.50 D in 32% of the operated eyes (Fig. 1E). At 5 years postoperatively, compared to 3 months postoperatively, 36% of the operated eyes had a postoperative SE Change of more than 0.50 D (Fig. 1F).

### Disk halo size

**Fig. 2:**
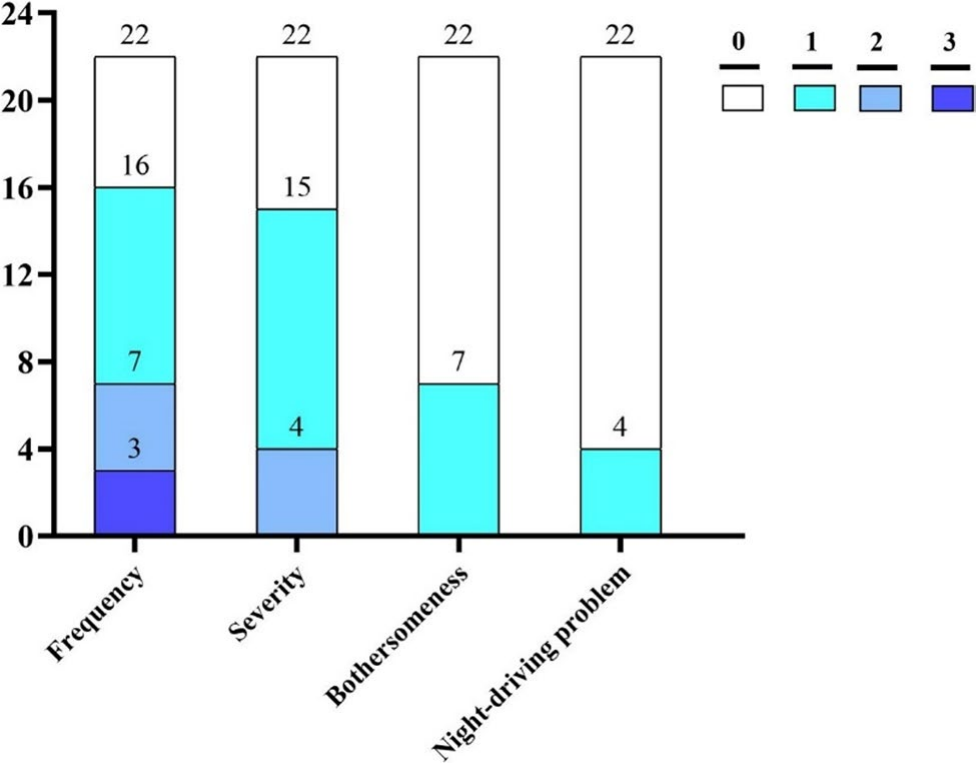
Time course of disk halo size after EVO implantable collamer lens (ICL) implantation

The glare values in myopic patients with ICL were 162.73 ± 87.37 arcmin, 122.95 ± 49.68 arcmin, 95.46 ± 38.97 arcmin, 79.55 ± 26.50 arcmin, and 72.96 ± 28.00 arcmin for the preoperative, 1-week, 1-month, 3-month, and 5-year postoperative periods, respectively, and the time effect was statistically significant (F = 22.685, *P* < 0.001, Fig. 2), Bonferonni two-way comparisons showed that the glare values at each postoperative time point were significantly lower than the preoperative values, and the differences were statistically significant (all *P* < 0. 01); the glare values at 1 month, 3 months and 5 years postoperatively were significantly lower than the 1 week postoperative values, and the differences were also statistically significant (all *P* < 0.001); and the difference between the glare values at 1 month, 3 months and 5 years postoperatively was not statistically significant (all *P* > 0.05).

### Patient-reported outcomes

**Fig. 3:**
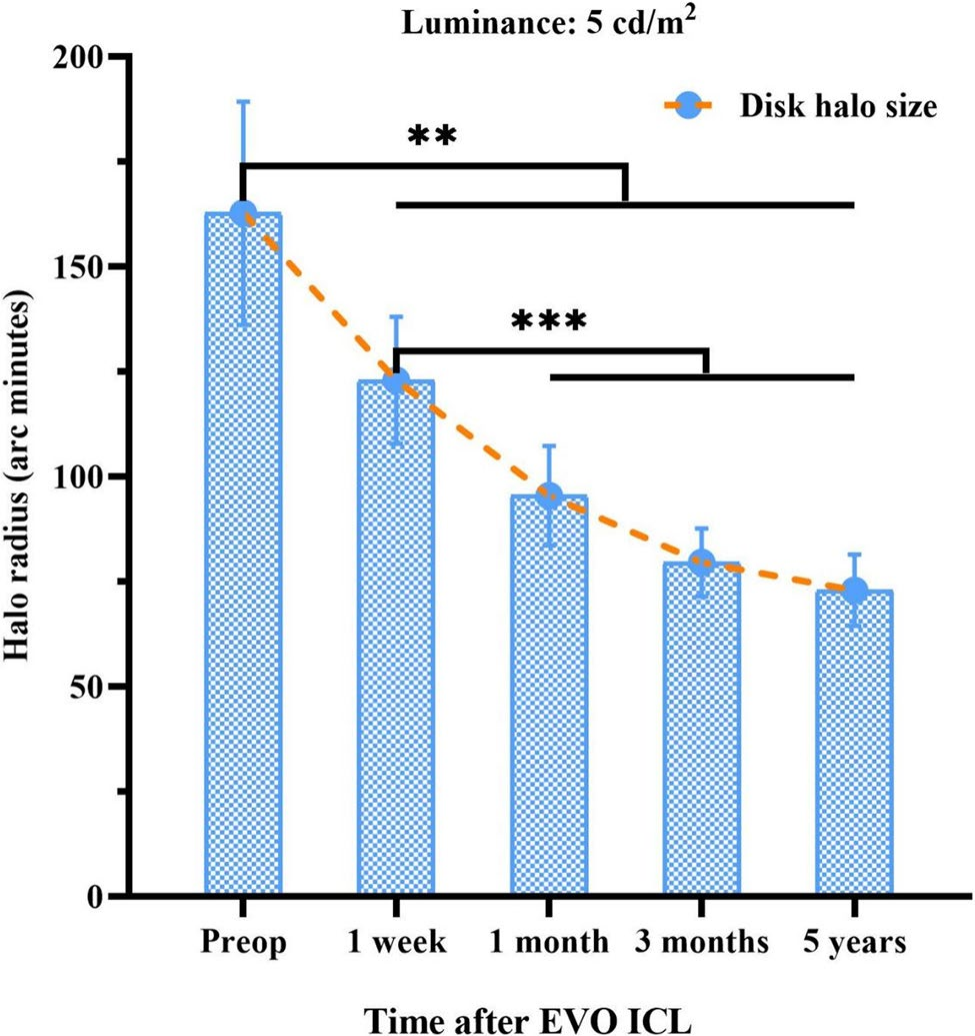
Glare symptom (frequency, severity and bothersomeness) and night-driving problem in patients 5 years after EVO implantable collamer lens (ICL) implantation

According to the results of the questionnaire, 72.7% (16/22) of the patients reported glare symptoms and 27.3% (6/22) of patients did not report any glare 5 years after ICL implantation, with 13.6% (3/22) of them reporting frequent symptoms. None of the patients, however, reported experiencing severe glare symptoms. Furthermore, glare bothersomeness were reported by 31.8% (7/22) of the patients but these bothersomeness were all mild in nature. Additionally, 18.2% (4/22) of patients reported mild impairment when driving at night (city driving, speed Limit 80 km/h). (Table 2; Fig. 3).

**Table 2:** Characteristics of patients who reported a problem with night-driving

Cases	Sex/age	Eye	Manifest refraction	UDVA (log MAR)	Dilation velocity (mm/s)	Maximum PD (mm)	ICL size (mm)
1	M/24	OD	+ 0.50/−3.00 × 155—1.2	0.1	2.72	3.78	13.2
		OS	−0.50/−1.25 × 180—1.2	0.1	2.35	4.17	13.2
2	F/38	OD	+ 0.75/−0.75 × 65 —1.5	−0.1	2.23	4.58	13.2
		OS	+ 0.25/−0.50 × 105—1.5	−0.1	2.15	4.51	13.2
3	F/38	OD	−0.25/−1.25 × 60 —1.2	0.1	1.72	4.17	13.2
		OS	−1.25/−0.75 × 95—1.0	0.3	1.70	4.91	13.2
4	M/23	OD	+ 0.50/−1.50 × 25—1.2	−0.1	4.08	6.34	13.2
		OS	−0.75/−1.00 × 25 —1.2	0.2	3.79	6.30	13.2

*UDVA* uncorrected distance visual acuity, *PD *pupil diameter, *ICL *implantable collamer lens

### Dynamic pupillometry results

Dynamic pupillometry at preoperative and postoperative time points of ICL showed that, except for the minimum pupil diameter, the time effects of the remaining parameters such as the amplitude of pupil contraction, contraction velocity, and dilation velocity, as well as the time effects of changes in the maximum and average pupil diameters, were all statistically significant (Table 3). The contraction velocity at 1 and 3 months, the dilation velocity at 3 month, the minimum pupil diameter at 1 week and 1 month were relevant to the vault (*P* < 0.05).

**Table 3:** Dynamic pupillometry data for EVO ICL-treated patients

Characteristics	Preop	Postop-1 w	Postop-1 m	Postop-3 m	Postop-5 y	F	*P*
Amplitude of contraction, mm	1.91 ± 0.28	1.61 ± 0.26	1.62 ± 0.26	1.61 ± 0.27	1.71 ± 0.54	6.192	0.000
Contraction velocity, mm/s	6.18 ± 0.67	5.01 ± 0.69	4.98 ± 0.74*	5.02 ± 0.80**	5.13 ± 0.69	22.831	0.000
Dilation velocity, mm/s	2.55 ± 0.73	2.07 ± 0.26	2.10 ± 0.37	2.02 ± 0.33**	2.44 ± 0.87	8.066	0.000
Maximum pupil diameter, mm	5.56 ± 0.85	5.33 ± 0.48	5.27 ± 0.52	5.22 ± 0.51	4.96 ± 0.59	5.634	0.000
Minimum pupil diameter, mm	3.25 ± 0.81	3.31 ± 0.40*	3.29 ± 0.41**	3.23 ± 0.34	3.05 ± 0.48	1.740	0.142
Average pupil diameter, mm	4.63 ± 0.89	4.52 ± 0.48	4.52 ± 0.47	4.45 ± 0.42	4.23 ± 0.58	2.793	0.027

*Relevance to the vault *P* < 0.05, ** *P* < 0.01

*ICL* implantable collamer lens, *Preop* preoperative, *Postop *postoperative, *w* week, *m* month, *y *year

Subsequent pairwise comparisons showed that: The amplitude of contraction was significantly reduced at 1 week, 1 month, and 3 months postoperatively compared to the preoperative period (all *P* = 0.001); it had mostly recovered by 5 years postoperatively (*P* = 0.081). Nevertheless, pairwise comparisons of all postoperative time points did not reveal statistically significant differences (*P* > 0.05). The pupil contraction velocity was significantly lower at all postoperative time points when compared to the preoperative period (all *P* < 0.001). However, there were no statistically significant differences (*P* > 0.05) found in pairwise comparisons of all postoperative time points following ICL. Pupil dilation velocities were significantly lower for the first week, month, and three months postoperatively when compared to the preoperative period (*P* < 0.01). However, the dilation velocities were mostly restored five years after surgery (*P* > 0.05). Pupil dilation velocities were significantly higher at 5 years postoperatively than the velocities measured at 1 week, 1 month, and 3 months (*P* < 0.05). No statistically significant difference was observed among the measured dilation velocities at 1 week, 1 month, and 3 months after surgery (*P* > 0.05). After 5 years of surgery, the maximum pupil diameter significantly decreased in comparison to the measurements taken before and one week after the surgery (*P* = 0.001 and 0.044 accordingly). The difference in maximum pupil diameter was not statistically significant when pairwise comparisons were made among the time points for preoperative, 1 week, 1 month, and 3 months after surgery (*P* > 0.05 for all). Five years after surgery, the average pupil diameter was significantly smaller than the preoperative values (*P* = 0.016), but no significant changes in average pupil diameter were observed at one week, one month, and three months after surgery (all *P* > 0.05). When comparing the postoperative time points with each other, no significant differences were found (all *P* > 0.05).

### ECD

The ECD of the ICL-treated eye was 3090.5 ± 251.5 cells/mm^2^, 2992.2 ± 293.1 cells/mm^2^, and 2819.5 ± 324.9 cells/mm^2^ before the surgery, three months, and five years after the surgery, respectively. A statistically significant time effect was observed (*P* < 0.05). No significant change was observed in ECD at three months postoperatively as compared to preoperatively (*P* > 0.05). However, a significant reduction in ECD was noticed both at five years postoperatively compared to preoperatively and at three months postoperatively (both *P* < 0.05). The mean rate of ECD loss was 8.77% at five years after ICL.

### Vault

The vault measurements taken at 1 month, 3 months and 5 years after ICL surgery were 662.0 ± 223.7 μm, 669.1 ± 265.7 μm and 545.0 ± 213.0 μm, respectively. We observed a statistically significant effect of time (*P* < 0.05). A significant decrease was observed at 5 years postoperatively in contrast to one month and three months postoperatively, respectively (both *P* < 0.05). However, there was no significant change observed at both one month and three months postoperatively (*P* > 0.05).

### Multivariate analysis

The multivariate analysis included the patient’s profile, ICL parameters, vault, and dynamic pupil parameter values. According to the GEE results, the patient’s pupil dilation velocity, maximum pupil diameter, and ICL size were positively correlated with the postoperative glare values, and were identified as the main influencing factors (all *P* < 0.01, Table 4).

**Table 4:** Multivariate analysis for glare in EVO ICL-treated patients

	B coefficient	95% CI	χ^2^	*P*
Slope	−1224.879	−2123.718, −326.040	7.134	0.008
Eye [right = 0, left = 1]	21.790	−18.325, 61.905	1.133	0.287
Gender [male = 0, female = 1]	20.003	−43.931, 83.936	0.376	0.540
Age	−1.692	−7.057, 3.673	0.382	0.536
Axial length	−19.834	−48.593, 8.925	1.827	0.176
Astigmatism	−11.931	−69.934, 46.071	0.163	0.687
Spherical equivalent	−22.316	−48.469, 3.838	2.797	0.094
UDVA	79.514	−379.115, 538.143	0.115	0.734
Amplitude of contraction	−107.484	−236.486, 21.519	2.667	0.102
Contraction velocity	15.782	−19.861, 51.425	0.753	0.385
Dilation velocity	39.997	16.448, 63.546	11.082	0.001
Maximum pupil diameter	122.599	39.568, 205.630	8.375	0.004
Minimum pupil diameter	−4.377	−118.358, 109.604	0.006	0.940
Average pupil diameter	−88.621	−200.314, 23.072	2.418	0.120
ICL size	104.298	37.938, 170.658	9.489	0.002
Sphere @ ICL	7.214	−18.238, 32.666	0.309	0.579
Astigmatism @ ICL	−13.830	−59.480, 31.819	0.353	0.553
Vault	0.056	−0.080, 0.193	0.651	0.420

*UDVA* Uncorrected distance visual acuity, *ICL *implantable collamer lens

## Discussion

ICL surgery has unique advantages in the correction of high and ultra-high myopia, but glare is still one of the most common nocturnal symptoms after ICL in myopic patients. In this paper, based on the observation of the efficacy of post-ICL surgery in myopic patients, we have, for the first time, used a combination of subjective and objective methods to assess the symptoms of long-term glare after ICL surgery for moderate to high myopia, and explored the main factors influencing post-ICL glare, which has some guiding significance for the clinical development of myopic ICL surgery.

The result showed that the efficacy index 5 years after ICL was below 1.0 while the safety index was above 1.0; among them, 14 eyes (SE< −8.88 D) experienced axial length elongation. Our team had also previously observed Changes in axial length in myopic patients 5 years after ICL [9], suggesting that preoperative counselling should pay attention to refraction in high myopia patients regarding axial length stability.

The glare test results show that the size of the disc halo begins to improve in patients 3 month after ICL and is maintained up to 5 years after surgery. This is the first direct measurement of long-term glare symptoms in patients after ICL implantation. This result is close to physiological values reported in previous literature [10] and measurements in myopic patients [7]. The difference between the glare values at 1 month, 3 months and 5 years postoperatively was not statistically significant (all *P* > 0.05), which is also consistent with our team’s previous observation that disc halo size can remain stable in the early postoperative period after ICL implantation [2]. In addition, the results of the questionnaire scale showed that of the patients who reported glare symptoms, 7 had mild bothersomeness. Four of these cases reported a mild effect - night driving behavior became conservative and cautious (Table 2). The reason for this may be due to decreased night vision in dark environments caused by mild myopia and astigmatism 5 years after surgery, or glare symptoms related to fast pupil displacement velocity and lager pupil diameter.

In this study, we found that the pupil contraction velocity decreased significantly at all time points after ICL implantation, whereas the amplitude of pupil contraction and the dilation velocity decreased significantly in the early postoperative period and had returned to preoperative levels by 5 years postoperatively. In vivo UBM studies have demonstrated contact between the ICL and the posterior iris surface [11, 12]. Mechanical contact between the ICL and the iris under high vault conditions as well as provocation may lead to a reduction in the amplitude and velocity of pupil contraction. Besides, this study showed that the early changes in the values of the above parameters are relevant to the vault, and the findings of Zhu et al. [13] in the early postoperative period after ICL implantation also corroborate our results. With the regression of the vault in the long-term period, the contact area between the ICL and the posterior iris surface decreases accordingly, and the amplitude of pupil contraction and dilation velocity may therefore gradually return to the preoperative levels. In addition, the present study also observed that the maximum pupil diameter decreased significantly 5 years after ICL surgery. On the one hand, mechanical irritation of ICL and the uveal tissue may have played a role, which causing a decrease in the ability of iris muscles [14, 15]; On the other hand, physiological change of pupil size and accommodation may have affected our findings [16, 17].

The results of this study show that pupil dilation velocity, maximum pupil diameter and ICL size are all positively correlated with post-ICL glare and are the main influences on post-ICL glare. This suggests that the larger the values of these 3 parameters, the more pronounced the patient’s postoperative glare symptoms may be. In the early postoperative period after ICL surgery, physical contact, and provocation between the ICL and the iris may cause changes in the values of pupillometric parameters, such as smaller dilation velocity and maximum pupil diameter. Therefore, preoperative pupil assessment and patient counseling were recommended. On the other hand, post-ICL glare is associated with intraocular scattering [18], and the objective scattering index (OSI) can be significantly reduced in the short-term postoperative period [19]. These changes may be the potential reason for the significant reduction in glare in the very early post-ICL period (within 1 month) in this study. While long-term reports are still scarce, Sanders et al. [20] reported that 3 years after ICL implantation in eyes with moderate to high myopia, there was no significant increase in the incidence of nocturnal visual symptoms such as glare and halos compared to the preoperative period. Chan et al. [21] found that 5 years after ICL for moderate to high myopia, 7.9% of the operated eyes reported symptoms of glare and halos.

Although the larger the ICL size, the greater the postoperative vault [22], the vault gradually decreases over time, reducing the contact area between the ICL and the iris and restoring the pupil dilation velocity. At the same time, the maximum pupil diameter decreases significantly with age. The combined effect of these changes may be the reason why long-term glare can remain relatively stable after ICL.

### Strengths and limitation

The combination of subjective and objective methods to assess long-term glare after ICL and the use of GEE to analyze its main determinants, making full use of longitudinal repeated measures data and improving statistical power, are the strengths of this paper. However, this paper still has weaknesses: for example, this study was affected by the COVID-19 pandemic and the time points of follow-up after ICL were relatively few and the actual gap between the last 2 follow-ups was more than 4 years. Besides, the study lacks a control group and the sample size was small. It is necessary to include more subjects and follow-up time points in future studies. In addition, the omission of the accommodation factors on the pupil is also a limitation.

## Conclusion

EVO ICL is safe and effective in correcting myopia. Post-operative glare is related to the pupil dilation velocity, maximum pupil diameter, and halo radius is improved in the early postoperative period, then remained stable. Most patients have no glare bothersomeness in the long term, some patients may even have their glare symptoms disappear. Only a few patients experience slight interference while nighttime driving. Even mild glare can impact quality of life in sensitive tasks like night driving.

## Data Availability

Data and materials are available upon request from the corresponding author at doctxiaoyingwang@163.com or doctzhouxingtao@163.com.
